# Corroded Bolt Identification Using Mask Region-Based Deep Learning Trained on Synthesized Data

**DOI:** 10.3390/s22093340

**Published:** 2022-04-27

**Authors:** Quoc-Bao Ta, Thanh-Canh Huynh, Quang-Quang Pham, Jeong-Tae Kim

**Affiliations:** 1 Department of Ocean Engineering, Pukyong National University, Busan 48513, Korea; qb.tabao@gmail.com (Q.-B.T.); bkdn06x3a@gmail.com (Q.-Q.P.); 2 Institute of Research and Development, Duy Tan University, Danang 550000, Vietnam; huynhthanhcanh@duytan.edu.vn; 3 Faculty of Civil Engineering, Duy Tan University, Danang 550000, Vietnam

**Keywords:** deep learning, vision-based approach, Mask-RCNN, bolt corrosion, image processing, bolted connection, steel structures

## Abstract

The performance of a neural network depends on the availability of datasets, and most deep learning techniques lack accuracy and generalization when they are trained using limited datasets. Using synthesized training data is one of the effective ways to overcome the above limitation. Besides, the previous corroded bolt detection method has focused on classifying only two classes, clean and fully rusted bolts, and its performance for detecting partially rusted bolts is still questionable. This study presents a deep learning method to identify corroded bolts in steel structures using a mask region-based convolutional neural network (Mask-RCNN) trained on synthesized data. The Resnet50 integrated with a feature pyramid network is used as the backbone for feature extraction in the Mask-RCNN-based corroded bolt detector. A four-step data synthesis procedure is proposed to autonomously generate the training datasets of corroded bolts with different severities. Afterwards, the proposed detector is trained by the synthesized datasets, and its robustness is demonstrated by detecting corroded bolts in a lab-scale steel structure under varying capturing distances and perspectives. The results show that the proposed method has detected corroded bolts well and identified their corrosion levels with the most desired overall accuracy rate = 96.3% for a 1.0 m capturing distance and 97.5% for a 15° perspective angle.

## 1. Introduction

Bolted joints serve to connect load-bearing members in various structural systems. During their life cycle, bolted joints are frequently exposed to different environmental conditions of moisture and air pollutants and therefore are susceptible to corrosion [1,2]. Thus, early detection of bolt corrosions should be regularly and periodically implemented to secure the safety and integrity of the bolted joints.

In the past few decades, many structural health monitoring (SHM) methods have been developed to gradually replace time-costly traditional visual inspections [3,4,5,6,7]. Researchers have attempted to monitor the health of bolted connections using contact-sensor-based methods, such as the acoustic-based method [8], wavelet-analysis-based methods [9], coda wave interferometry [10], piezoelectric-active-based methods [1], electromechanical-impedance-based methods [11,12], fiber Bragg gratings (FBGs)-based methods [13,14]. However, those methods often require expensive and precise instruments to record the damage-sensitive responses from a bolted joint. They are also ineffective when implementing a large bolted joint and in field experiments. Although the wireless-based damage bolt monitoring sensors [7] have been designed to be flexible, energy-saving, and promising for realistic structures, maintaining and protecting those sensors under hazardous environment conditions are the dilemma issues. Besides, the data recorded from the sensors and actuators are affected by environmental factors, leading to a certain difficulty in damage-feature extraction. Recently, the vision-based SHM approach has been receiving significant interest from the research community [5]. This technology has the potential to solve the inherent issues of sensor-based techniques. In brief, the vision-based approach offers the following advantages: a simple setup and operation, non-contact sensing technology, immune to temperature and humidity changes, suitable for SHM of large structures.

Due to those promising features, the machine learning and vision-based approaches have been applied for SHM of steel bolted joints [15,16,17,18,19,20,21,22]. Several vision-based bolt-looseness monitoring techniques were developed on the basis of the region-based convolutional neural network (RCNN) or Faster RCNN [23,24,25]. Pan et al. [26] proposed a detect-track method based on the YOLOv3-tiny [27] and Kanade–Lucas–Tomasi (KLT) algorithm [19] to track the rotation of clean bolts under lab-scale light conditions. Cha et al. [17] also proposed a vision-based faster RCNN model for detecting multi-damage in civil structures (i.e., steel corrosion, delamination, etc.). Moreover, Chun et al. [28] used a deep learning-based image captioning technique to explain bridge damage types (e.g., corrosion, cracks). However, most of the studies have focused on stainless bolts and only a few studies have developed a vision-based method for corroded bolt detection. Recently, Ta et al. [24] proposed an RCNN-based corroded bolt detector and verified it on a lab-scale bolted joint of a steel girder. Despite the promising result, the proposed detector was developed to identify two classes, clean and fully rusted bolts, and its performance for detecting partially rusted bolts is still questionable. Thus, there is a need to develop an alternative deep-learning-based method for detecting early-corroded bolts in the steel joint. Mask-RCNN is the state-of-the-art algorithm for object segmentation and is developed on top of Faster RCNN. One of the major advantages of the Mask-RCNN model is that it can return both the bounding box for each object and its instance segmentation. Thus, the use of the Mask-RCNN model could allow for the further quantification of the severity of corrosion damage on bolts at the pixel level.

The performance of a neural network depends on the availability of datasets, and most deep learning techniques lack accuracy and generalization when they are trained using limited datasets. With the aim of advancing the perception of deep learning models, many studies have developed computer graphics to synthesize realistic virtual models that support creating the training graphic datasets. Several deep learning models trained on synthesized datasets have recently demonstrated an outstanding performance when tested on realistic datasets [29,30,31]. Regarding bolted joint monitoring, Pham et al. [29] built a 3D graphical model of a bolted plate to generate high-quality images for training an RCNN-based bolt detector. The proposed model achieved fairly good detection results compared to the model trained on realistic data. Recently, Yang et al. [27] have developed a graphical model with bolt-loosening marks to create synthetic datasets for training YOLO-based deep learning models. They investigated bolt-looseness detection under different environmental conditions and achieved pretty good results. However, the graphical models from those studies, consisting of only clean bolts, can be easily created with less time. In the case of modeling a corroded bolted joint, it will take a long time to build a realistic graphical model and render it considering actual environment behaviors. The process of manual label annotation for the training dataset from those studies was also incredibly timewasting, especially in the case of distributed labeling objects within a narrow complex background.

The objective of this study is to develop a deep learning model for identifying early-corroded bolts in steel joints and to present a data synthesis technique to autonomously generate training datasets. Firstly, a Mask-RCNN-based corroded bolt detector is proposed. The Resnet50 integrated with the feature pyramid network is used as the backbone for feature extraction in the detector. Next, a four-step procedure is proposed to autonomously generate three datasets: clean bolt (Bolt), partially corroded bolt (PCBolt), and fully corroded bolt (FCBolt), along with their corresponding masks. Thirdly, the generated datasets are used to train the Mask-RCNN-based corroded bolt detectors. Finally, the robustness of the method is demonstrated by detecting corroded bolts in a lab-scale steel structure under varying capturing distances and perspectives.

## 2. Mask-RCNN-Based Corroded Bolt Detector

### 2.1. Architecture of the Detector

The Mask-RCNN model is built based on the Faster RCNN model for instant object segmentation with three primary changes: (1) a Resnet50 backbone is integrated with the feature pyramid networks (FPN) to generate better-scaled features; (2) RoiAlign layers are used instead of the RoiMaxPooling layers to provide more sub-pixel levels than RoiMaxPooling layers; and (3) a mask branch for pixel-level object segmentation is attached to generate pixel masks. 

**Figure 1:**
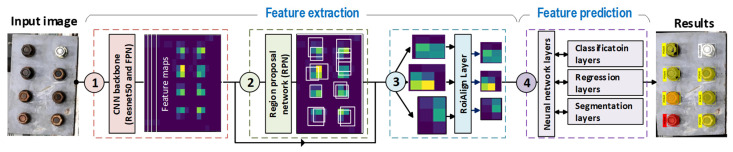
Overall architecture of the Mask-RCNN-based corroded bolt detector.

According to the previous studies [32,33] a Mask-RCNN model is developed for identifying corroded bolts in bolted connections, as shown in Figure 1. The Mask-RCNN-based corroded bolt detector can automatically identify bolts in the input image, classifying them into as clean bolts (ClBolt), corroded bolts (PCBolt) or fully corroded bolts (FCBolt), and deliver corresponding segmentation maps (e.g., a pixel mask) with individual identities. This could help to provide a faster process for retrieving object information, even with overlapped objects in a similar category, as compared to semantic segmentation tasks [34].

As illustrated in Figure 1, the Mask-RCNN architecture includes two primary modules: feature extraction and feature prediction. In the first module, a Resnet50-Feature Pyramid Network (FPN), Region Proposal Network (RPN), and RoiAlign layers are sequentially designed to extract plausible features information from the input image. The feature prediction module is a series of neural network layers such as classification, regression, and segmentation that take responsibility for predicting class names, bounding boxes, and the pixel masks of the bolt features, respectively. The classification and bounding box regression offset, as the detection branch runs parallel with the segmentation branch. In the following, the feature extraction and the feature prediction modules are described in detail.

Detection results from each category are marked by the different colors. For example, clean bolts (ClBolt) are assigned by a white color at the bounding boxes and masks, whereas the partially corroded bolts (PCBolt) and fully corroded bolts (FCBolt) are marked by yellow and red colors, respectively.

#### 2.1.1. Feature Extraction Module

##### Resnet50-FPN

**Figure 2:**
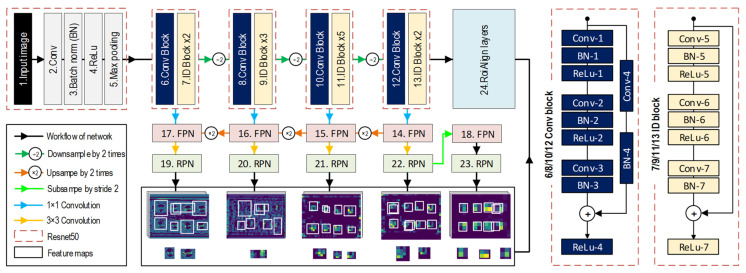
Resnet50-FPN is equipped with RPN and RoIAlign layer for feature extraction.

The architecture of the Resnet50-FPN is depicted in Figure 2 [35,36]. The detailed visualization of the Resnet50-FPN shows the first 13 layers are of the Resnet50, and the five next layers (i.e., 14, 15, 16, 17, and 18) are of the FPN net.

Different from other networks, the Resnet50 network [35], a very deep network, is built based on Convolutional (Conv) and Identity (ID) blocks. These blocks are stacked and repeated to deal with the vanishing gradient problem in the learning process when the gradient is extremely small [35]. The convolution blocks are used to skip the connection in case of unmatching between the input and output. Each fusion block of the Resnet50 is built with the aim of halving the feature map sizes and doubling the number of feature maps. The Resnet50 was adopted for extracting feature maps of bolts due to its good performance as the winner in the ImageNet Large Scale Visual Recognition Challenge (ILSVRC) competition in 2015 [37]. In this study, the ResNet50 was modified to fit the Mask-RCNN for feature learning of bolt images. 

As fundamental components of an object recognition system [36], the FPN layers are combined with the Resnet50 for detecting bolts at different scales. The maps from the ResNet50 apply an operation of 1 × 1 convolution before passing to the FPN layers, as shown in Figure 2. These 1 × 1 convolution layers not only help to shrink the number of feature channels, reduce computational cost, and remove unimportant features, but also support finding out many other complex hidden features from the feature maps extracted from the Resnet50 network without changing the size of the features’ width and height. According to the increasing orders of the FPN layers, an up-sampling operation by two times is used to build each pyramid of the bolt features excepted for the 18th FPN layer. 

##### RPN

As the second module, the region proposal network (RPN) layers (see Figure 1) receive the feature maps from the FPN layers to directly create region proposals based on anchor boxes and scaled rates instead of using an external algorithm such as edge boxes [38], which makes the network faster and better at learning from data [33]. To obtain proposal boxes from the RPN layers, the maps continue being subjected to an operation of 3 × 3 convolution. It is noted that only the feature maps of the 18th FPN layer were generated by subsampling from the feature maps in the 22nd RPN layer with a stride of 2. Visualization of five levels of feature maps with anchor boxes from the RPN layers is shown in Figure 2.

##### RoiAlign Layer

The third module is the RoiAlign layers. They receive feature boxes and scale these features to a uniform size. By using an interpolation of feature calculation, the RoiAlign layers (the 24th layer) are used in Mask-RCNN instead of the RoiMaxPooling layers because they accurately provide sub-pixel levels better than RoiMaxPooling layers [32].

Table 1 shows layers and operators of modified Resnet50. It includes an input image layer (Input), convolution layers (Conv), a batch normalization layer (Batch norm), an activation function layer (ReLu), max-pooling layers (Max pool), four Conv blocks, and twelve ID blocks. The details of the FPN, RPN, and RoiAlign layers can also be found in [32].

#### 2.1.2. Feature Prediction Module and Loss Functions

The feature prediction module of Mask-RCNN (see Figure 1) is constructed from the Faster-RCNN’s feature prediction layers (e.g., classification and box regression layers) and the object segmentation layer. These layers receive scaled features from the RoiAlign layers and work in parallel to output the class, bounding box, and pixel mask of the objects (e.g., bolts).

The classification layer contains fully connected (FC) layers, and the final FC layer outputs four-class types, ClBolt, FCBolt, PCBolt, and BG. The classification loss function of the classification layer is defined in Equation (1), in which $p_{i}$ is the probability value of the $i^{th}$ anchor box, and $p_{i}^{*}$ is the probability value of the true class that is decided by the IoU index [32].

(1) $$L_{class}(p_{i},p_{i}^{*})=-log[p_{i}p_{i}^{*}+(1-p_{i}^{*})(1-p_{i})]$$

The box regression layers aim to generate bounding boxes covering the objects. This branch similarly operates as RPN layers: it refines the location and size of the bounding box. Equation (2) shows the bounding-box loss function ($L_{box}$), where $t_{i}$ and $t_{i}^{*}$ contain four components: the first two components represent the translation direction, and the others represent the scaling direction, as shown in Equations (3) and (4), respectively. The indexes x, y, w, h are the x coordinate, y coordinate, x-direction length, and y-direction length of the predicted bounding box, respectively. The indexes marked “*” refer to the coordinates of the ground truth box, and those marked “a” refer to the coordinates of the anchor box.

(2) $$L_{box}(t_{i},t_{i}^{*})=\left\{ \begin{array}{l} 0.5{\left(t_{i}-t_{i}^{*}\right)}^{2},\;if\left|x\right|<1\\ \left|x\right|-0.5,\;if\left|x\right|>1 \end{array}\right.$$

(3) $$t_{x}=\frac{x-x_{a}}{w_{a}},t_{y}=\frac{y-y_{a}}{h_{a}},t_{w}=log\frac{w}{w_{a}},t_{h}=log\frac{h}{h_{a}}$$

(4) $$t_{x}^{*}=\frac{x^{*}-x_{a}}{w_{a}},t_{y}^{*}=\frac{y^{*}-y_{a}}{h_{a}},t_{w}^{*}=log\frac{w^{*}}{w_{a}},t_{h}^{*}=log\frac{h^{*}}{h_{a}}$$

The mask segmentation layers are fully convolutional layers, and they take responsibility for producing pixel-level masks for each bolt using four consecutive 3 × 3 convolutional layers, a 1 × 1 deconvolutional layer, and a 1 × 1 convolutional layer. This branch’s architecture is specified in the Mask-RCNN paper by He et al. [32]. The output mask of the branch is modified to 4, which is equal to the number of classes in the classification branch. Equation (5) shows the mask loss function ($L_{mask}$). $L_{mask}$ is defined as the average binary cross-entropy loss for positive region of interests (RoIs), which has the IoU index overlap be equal to or larger than 0.5. The value $x_{i}$ is the $i^{th}$ pixel predicted in positive RoIs, the value $b_{i}$ is the correct $i^{th}$ pixel from the ground truth in positive RoIs, and N is the number of pixels in positive RoIs. The value of $y_{i}$, $a_{i}$ are shown in Equations (6) and (7), respectively.

(5) $$L_{mask}=-\frac{1}{N}[y_{i}ln(a_{i})+(1-y_{i})ln(1-a_{i})]$$

(6) $$y_{i}=\frac{1}{\left(1+e^{-x_{i}}\right)}$$

(7) $$a_{i}=\frac{1}{\left(1+e^{-b_{i}}\right)}$$

The total loss function during training of the Mask-RCNN model [32] for each anchor is shown in Equation (1). The representative loss value ($L_{loss}$) is calculated by the sum of the classification loss ($L_{class}$), bounding-box loss ($L_{box}$), and mask loss ($L_{mask}$).

(8) $$L_{loss}=L_{class}+L_{box}+L_{mask}$$

### 2.2. Evaluation Metrics

**Figure 3:**
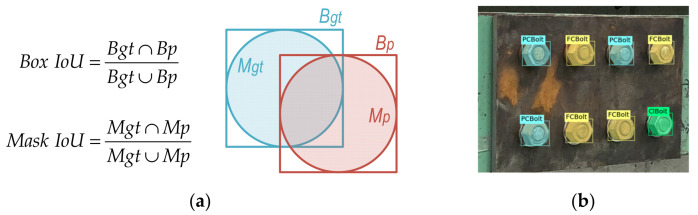
Definition of intersection over union: (**a**) Intersection over union (IoU); (**b**) Predefined ground truth.

Intersection over union (IoU) indices are used to calculate accuracy and error rates [39]. The concept and visualization of the IoU calculation are shown in Figure 3a. The IoU indexes indicate the proportion of overlapping areas between the predicted and ground truth values. The overlapping rate that is larger than 50 percent is considered the correct object. The Box IoU index is used to measure how well the object classes/categories are correctly classified, and the results of bolt classification are expressed by fusion matrix charts (see Section 5.1). As shown in Figure 3a, the Box IoU is the intersection between the box’s ground truth (Bgt) and the box’s prediction (Bp) over their union. The Mask IoU index is the intersection of the mask’s ground truth (Mgt) and the mask’s prediction (Mp) over their union, and it is used to calculate the mean average precision (mAP). The Mask IoU is used for the mAP calculation because the overlapping of bounding boxes is a poor approximation of object forms.

As shown in Equation (9), the Precision metric, the so-called true-predictive rate, is the true positives (TP) ratio over TP and false positives (FP), and the false-discovery rate is the FP ratio over TP and FP. The Recall metric, the so-called sensitivity or the true-positive rate, is denoted as the ratio of TP over TP and false negatives (FN), and the false-negative rate is the FN ratio over TP and FN. The BF score, the so-called contour matching score, measures the similarity between the predicted pixel segmentation in the prediction and the true pixel segmentation in the predefined ground truth. As depicted in Equation (9), the BF score is the harmonic mean of the Precision and Recall values and is calculated via the Mask IoU index.

Average precision (AP) represents the ability of the bolt detector to perform prediction tasks and figure out the corresponding objects accurately. It is noted that the P-R curve shows the variation of recall values at various levels, and the integration of areas under the corresponding P-R curve is the AP value. For a test image, the calculation of the AP for all categories/classes is shown in Equation (10), in which P is the Precision of the image, Δr is the change of the Recall, and N is the total number of divided segments. The mAP (see Equation (11)) is the index to assess the power of the Mask-RCNN network, and it averages the sum of the $AP_{i}$ value, in which i stands for the *i^th^* test image, and the q index indicates the total images used (e.g., ten images for each case).

Figure 3b shows an example of the predefined ground truth bolts for an 8-bolt splice connection, in which a clean bolt is labeled as “ClBolt”, three bolts are assigned as “PCBolt”, and the other ones are “FCBolt”.

(9) $$Precision=PositivePredictiveRate=\frac{TP}{TP+FP},\;FalseDiscoveryRate=\frac{FP}{TP+FP}\;Recall=TruePositiveRate=\frac{TP}{TP+FN},\;FalseNegativeRate=\frac{FN}{TP+FN}\;BF=2\times \frac{Precision\times Recall}{Precision+Recall}$$

(10) $$AP=\sum_{k=1}^{N}P(k)\Delta r(k)$$

(11) $$mAP=\frac{1}{q}\sum_{i=1}^{q}AP_{i}$$

## 3. Synthesized Data Generation and Training Process

### 3.1. Synthesized Data Generation

To overcome the issue of lacking training data, some researchers have synthesized the imagery datasets using synthetic environments with realistic image features [40,41,42,43]. The main idea is to crop the target objects and blend them with the backgrounds through transformation algorithms during the image blending process. The changes in the background not only reduce noise features from the original data but also enrich the training data. This strategy allows the simulation of target representations under a diversity of many complex conditions, which is hard to obtain by using 3D graphical models [29] or current data argumentation techniques [44]. The speed of the label annotation can be significantly enhanced by applying autonomous blending algorithms. 

**Figure 4:**
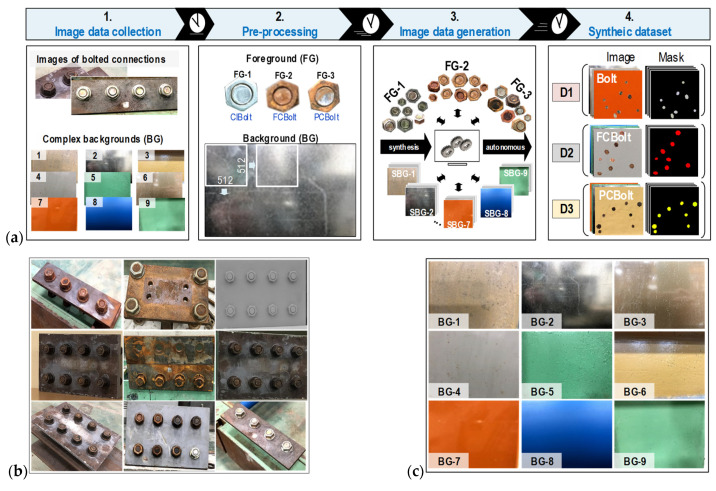

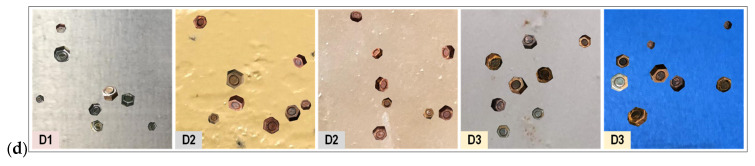
Synthetized data generation for the Mask-RCNN: (**a**) The four-step synthesis process of training data; (**b**) Examples of bolted connection; (**c**) Examples of complex backgrounds; (**d**) Examples of synthesized images.

To overcome the issue of limited training data and to enhance the robustness and generality of the Mask-RCNN-based corroded bolt detector for varying backgrounds (BG), a four-step synthesized dataset generation process is proposed in Figure 4a. The proposed process includes four main steps: (1) image data collection; (2) image pre-processing; (3) image data generation; and (4) synthetic dataset. This process can autonomously synthesize training datasets of the clean bolt (ClBolt), fully corroded bolt (FCBolt), and partially corroded bolt (PCBolt), along with their corresponding masks.

In the first step, 577 raw images, including 568 bolted connection images (see Figure 4b) and nine different background (BG) images (see Figure 4c), are collected from various steel structures. The size, color, and format type of the collected images are shown in Table 2. The 568 real-world images of bolted connections are captured by an iPhone X’s dual cameras at 1–1.5 m distances, 0–30° perspective distortions, and a roughly 154 Lux light intensity. The captured images have a 3024 × 4032 × 3 resolution and contain many levels of corroded bolts (from fully corroded to clean). Afterwards, the excess backgrounds were removed from the raw images. To feed the Mask-RCNN model, the width-cropped images are rescaled to 640 pixels, while their height follows the rate of the cropped width divided by 640. It is noted that the rescaled image size for the training image generation of the Mask-RCNN model follows the general image size of the COCO dataset [45]. The nine BG images are shot at a distance of around 0.2 m on different steel structures with the same resolution. The contents inside the BG images are complex, with different possessing properties such as colors, shadows, brightness, dirty spots, roughness, and random-noise points.

In the second step, the FG bolt images are cropped out with respect to the six edges of the bolt surfaces and then grouped into three groups, namely, FG-1 with 566 ClBolt images, FG-2 with 332 FCBolt images and FG-3 with 865 PCBolt images. The size of the FG images ranges from 32 × 42 × 3 to 173 × 187 × 3 pixel resolutions. Afterwards, the BG images are divided into sub-background (SBG) patches. Each BG image produces 46 SBG patches with the same size of 512 × 512 × 3. As seen in Table 2, the color and format of the FG images are RGB-alpha (RGBA) and “.png”, and those of the SBG images are red-green-blue (RGB) and “.jpeg”, respectively. The RGBA color is an extension of the RGB color, in which A (alpha) represents the level of transparency [46]. The “.png” format is selected for the FG bolt images because it supports transparent backgrounds, which means that when the alpha is equal to zero, the transparent background of the bolt image will be no color. Moreover, the image format “.png” could not be broken when modifying or mixing with other image formats [46].

In the third step, the domain randomization (DR) approach [47] was used to randomly combine the FG and SBG images for generating synthetic datasets. To form a synthetic image, an FG image is randomly orientated to a randomly-investigated background domain. This is a strong point of the synthesized datasets with regards to quality and environment compared to real-world datasets. To make data much larger in quantity [48] and reduce overfitting status [49], the FG bolts are randomly applied by the rotation, scale and brightness transformation algorithms in the respective ranges (0–360°], (0.6–1.0] and (0.7–1.2] during the image blending process. The ResNet50, which is a very deep learning network, is used to build the Mask-RCNN-based corroded bolt detector. To secure the success of the training process and enhance the performance of the CNN backbone and the feature prediction, a data argumentation technique was utilized as recommended in a few studies [44,50,51]. The FG address of the synthetic images, such as identity (ID) number, category/class name, and contour position, are saved in the “.json” formatted file, and the corresponding masks are automatically created from the saved file. 

In the final step, three synthetic datasets are autonomously created, including D1 (ClBolt), D2 (FCBolt), and D3 (PCBolt). Each dataset contains 1875 synthetic images of 512 × 512 × 3 pixels with 1875 corresponding annotated masks. All processing and image data generation processes use the python programing language (version 3.8). Figure 4d shows some examples of generated bolt images.

### 3.2. Training Process

The training process was conducted by a desktop computer system (GPU: GTX 2080 Ti 11G, CPU: Intel i9-9000KF 3.6 GHz, RAM: 64 GB) using python programing language (version 3.8) and the supporting libraries tensorflow [52], karas [53], and opencv [54]. The Mask-RCNN-based corroded bolt detector is trained on the synthesized datasets D1, D2 and D3 to identify ClBolt, FCBolt, and PCBolt, respectively. The training and validation images are randomly selected in each dataset, following the rate of 80% and 20%. Thus, the number of training and validation images is 4500 and 1125, respectively. The training algorithm is the stochastic gradient descent with momentum (SGDM) with a momentum of 0.9, a weight decay of 0.0005, and 1000 iterations per epoch. During the learning process, the IoU ratio is specified in the range of (0.8–1.0] for positive training samples. The anchor box of the RPN is defined with five sizes of (16; 32; 64; 128; 256] and three scales of (0.5; 1.0; 2.0]. With those setups, the bolt feature sizes (e.g., width × height) can be detected by down-sampling to an 8 × 8 pixel resolution and up-sampling to 384 × 384 pixels. Therefore, in the case of bolts with different sizes, the proposed Mask-RCNN model is still able to detect corroded bolts. For faster convergence and time-saving, the pre-trained ResNet50 is used [35]. To investigate the convergence of loss functions, the detector was run for 50 epochs with different learning rates: 10-4 (1st–30th epoch) and 10-5 (31st–50th epoch). The learning time of the detector was about 381 min.

**Figure 5:**
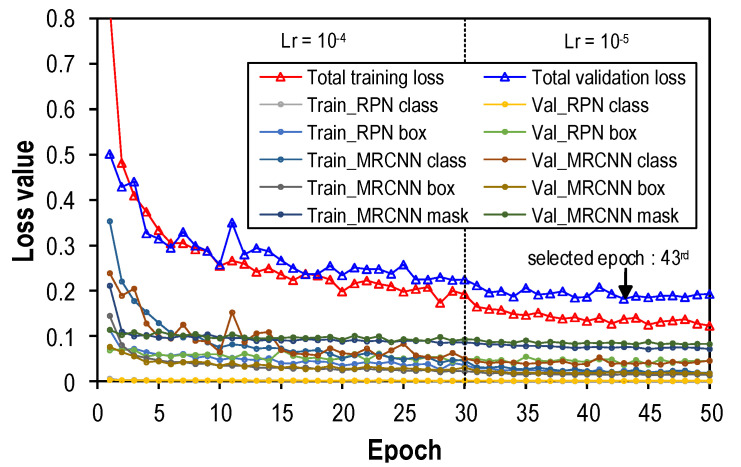
Results of training and validating the Mask-RCNN corroded bolt detector.

Figure 5 illustrates the training and validation results of the detector. The total loss values (including the training and validation loss) are summed up from the loss components: RPN class, RPN box, Mask-RCNN class, Mask-RCNN box and, Mask-RCNN mask [32]. In general, the loss value of the detector was sharply reduced in the first five epochs, followed by a gradual fall up to the 40th epoch, and then remained stable through the end of the learning process (50th epoch). The total training loss of the selected detector is approximately 0.13 at the 43rd epoch, which corresponds to the lowest validation loss of nearly 0.18.

As observed in Figure 5, there are sudden drops in the total loss lines at the 30th epoch due to a 10-time decrease in the learning rate, from 10^−4^ to 10^−5^. After the first 43 epochs, those loss lines are quite stable until the end of the learning process. Thus, the learning rate of 10^−5^ should be considered an optimal value for training the Mask-RCNN corroded bolt detector.

## 4. Experiments on Bolted Girder Connections

**Figure 6:**
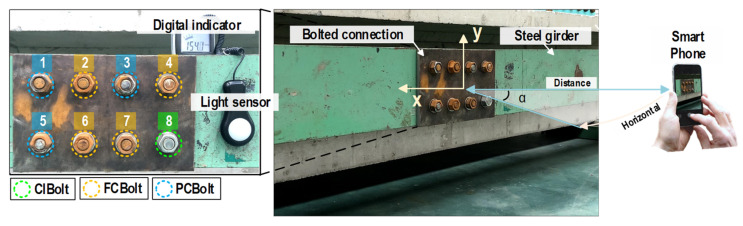
Lab-scale steel girder connection with clean and rusted bolts.

The corroded bolt detector was experimentally evaluated for identifying the early-corroded bolts in a lab-scale steel girder. The structure was constructed from two steel H-girder segments connected by bolted flanges, as shown in Figure 6. Details about the geometrical parameters of the test structure can be found in [24]. The target joint has eight pairs of bolts and nuts (Bolt 1–Bolt 8). Among them, Bolts 2, 4, 6, and 7 are fully corroded bolts (FCBolt), Bolts 1, 3, and 5 are partially corroded bolts (PCBolt) with different corrosion levels, and Bolt 8 is a clean bolt (ClBolt). It is noted that the corrosion was artificially created on the bolts and nuts using an acid solution (10% HCl) and by putting them under a wet condition. It is noted that the distribution of corrosion spots on the bolts and nuts is random to simulate a realistic situation. Clean washers were also inserted into Bolts 6 and 7 to simulate uncertainties. 

**Figure 7:**
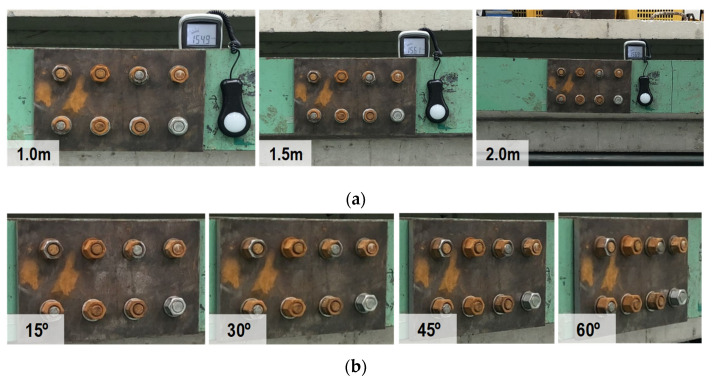
Lab-scale experiments under different conditions of distance and perspective distortion: (**a**) Various capture distances; (**b**) Various horizontal perspective distortions.

The camera of an iPhone X (with the following specs: 12 MP, wide-angle f/1.8 aperture, telephoto f/2.4 aperture) was used to shoot the image of the bolted connection with a 3042 × 4028 pixel resolution. During the experiment, the light intensity at the bolted joint was controlled at around 154~157 lux (measured by a digital light meter-GILTRON GT 1309, as shown in Figure 6).Two capturing conditions were investigated, including varying capturing distances and perspective angles. For each case, 10 images were captured. Figure 7a shows the bolted connection images captured under a distance of 1.0 m, 1.5 m, and 2.0 m with no perspective distortion. Figure 7b shows the bolted connection images captured under a perspective angle of 15°, 30°, 45°, and 60° with a fixed distance of 1.5 m. In total, 30 images were captured under different distances, and 40 images were shot under different perspective angles.

## 5. Corroded Bolt Detection Results

### 5.1. Corroded Bolt Detection under Various Capturing Distances

**Figure 8:**
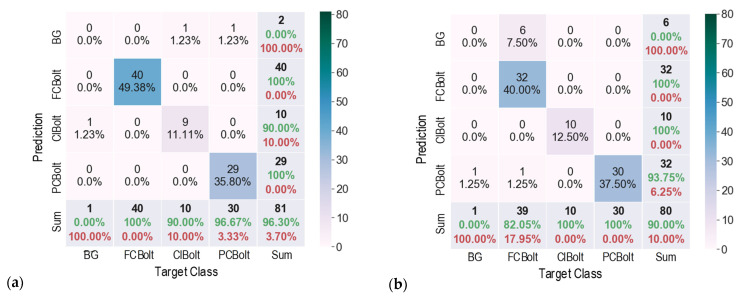

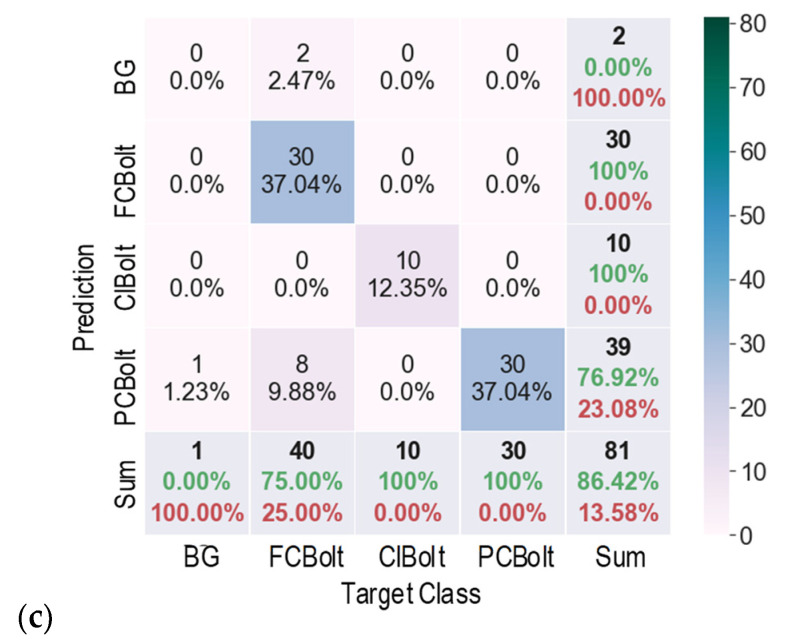
Fusion matrix chart for different capturing distances: (**a**) 1.0 m; (**b**) 1.5 m; (**c**) 2.0 m.

For different capture distances, the accuracy of the corroded bolt detector was evaluated using fusion matrix charts, as shown in Figure 8. It is noted that each cell in a matrix chart contains the number of observations and their percentage. The diagonal cells (from left to right) are observations that are correctly classified. The off-diagonal cells represent incorrectly classified observations. In the “Sum” column, each cell shows orderly the sum of the number of observations, the percentage of correctly predicted observations (in green) that belong to each class, and the percentage of incorrectly predicted observations (in red) belonging to each class. In the Sum row, each cell shows orderly the sum of the number of observations, the percentage of correctly classified observations, and the percentage of incorrectly classified observations. The bottom-right cell of the chart indicates the overall accuracy (in green).

**Figure 9:**
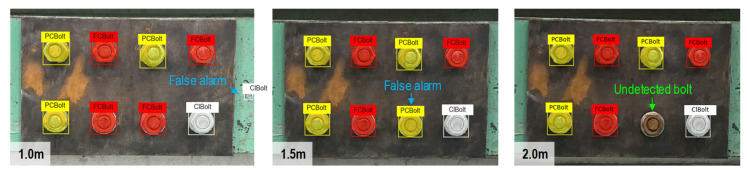
Visualization of the corroded bolt detection results at different capturing distances.

As shown in Figure 8a, for a capturing distance of 1.0 m, the detector precisely detected FCBolt and PCBolt, and 90% of ClBolt was correctly classified. The percentage of correctly classified observations for PCBolt reached about 96.67%. The overall accuracy of the detector was 96.3% for 1.0 m capturing distance. For a capturing distance of 1.5 m, as depicted in Figure 8b, ClBolt and FCBolt were correctly predicted. The PCBolt prediction accounted for an accuracy of 93.75%, and the overall accuracy of the detector was 90%. In the case of 2.0 m capturing distance, as shown in Figure 8c, the prediction accuracy was 100% for ClBolt and FCBolt and 76.92% for PCBolt, and the overall accuracy accounted for only 86.42%. It is found that ClBolt and FCBolt were identified with high accuracies (over 90%) for a capturing distance of up to 2.0 m, and PCBolt was classified as accurate for a capturing distance of up to 1.5 m. The representative results of corroded bolt identification for different capturing distances are visualized in Figure 9. As seen in Figure 9, there was a false alarm out of the joint at 1.0 m of distance. The reason is that the contents of the background area appear similar to the ClBolt’s shapes, which made the model incorrectly detect the mark as ClBolt. At a 2.0-m distance, the proposed model was unsuccessful in recognizing a corroded bolt. This could be caused by the reduction in the resolution of the bolt’s image. As the capturing distance is increased, the resolution of the bolt’s image is decreased. As a result, the bolt features in the captured image are altered, leading to the reduced accuracy of the corroded bolt detector.

**Figure 10:**
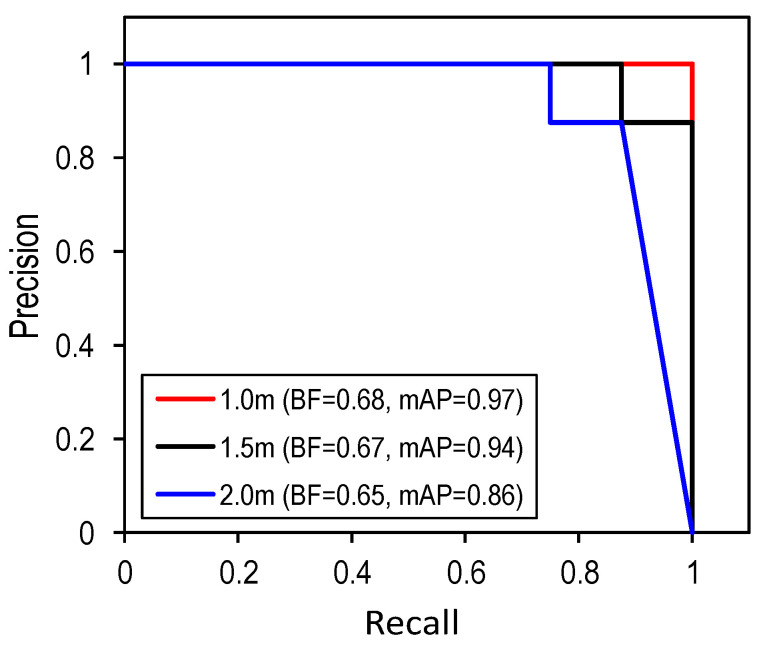
P-R curve, BF and mAP for different capturing distances.

The P-R curves were plotted in Figure 10. The BF and mAP values of the detector were computed for the three capture distances. As the capturing distance was increased, the BF and mAP values decreased, indicating a reduction in the prediction accuracy of the detector. The reason could be that the resolution of the captured images decreased with an increased capturing distance. Particularly, the BF value obtained at a 1.0-m and 1.5-m distance were 0.68 and 0.67, respectively, while that at 2.0 m was 0.65. The mAP value was 0.97, 0.94, and 0.86, corresponding to 1.0 m, 1.5 m, and 2.0 m. Conclusively, the partially corroded bolts (PCBolt) were well classified by the proposed detector, although there still were some false alarms.

**Figure 11:**
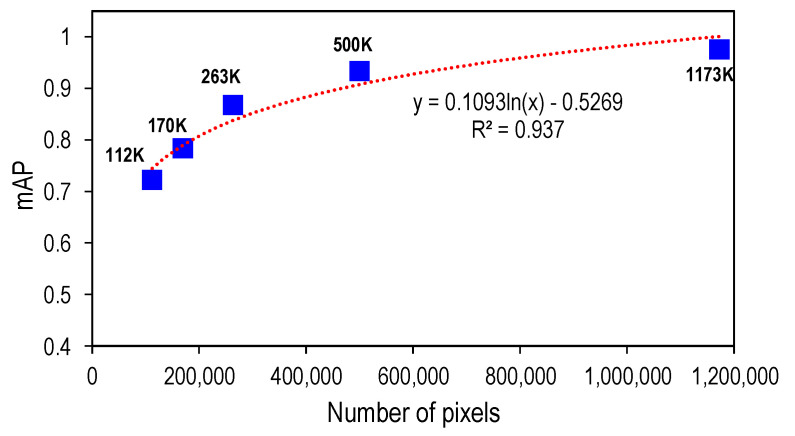
Resolution effect of the pixel number on the performance of Mask-RCNN-based corroded bolt detector.

The effect of the pixel resolution number on the performance of the Mask-RCNN-based corroded bolt detector is plotted in Figure 11. It is shown that the accuracy of the model decreases along with the decrease in the resolution. When the resolution is reduced to less than 263K pixels, the mAP of the detector dropped significantly (see Figure 11).

### 5.2. Corroded Bolt Detection under Various Perspective Distortions

**Figure 12:**
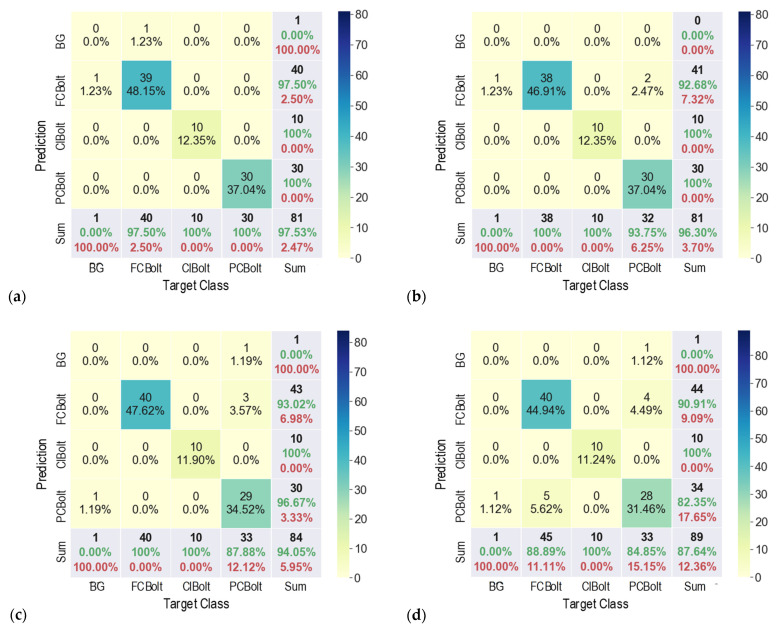
Fusion matrix chart for different capturing angles: (**a**) 15°; (**b**) 30°; (**c**) 45°; (**d**) 60°.

The results of corroded bolt classification under varying capturing angles (at 1.5 m capturing distance) are shown in Figure 12. Overall, the accuracy of the detector was reduced with an increased perspective angle. For a capturing angle of 15° (as seen in Figure 12a), the proposed detector identified ClBolt and PCBolt in the bolted joint with 100% accuracy, and the prediction for FCBolt was 97.50% accounted for. At the 30° capturing angle (as seen in Figure 12b), the prediction accuracy was slightly reduced to 92.68% for FCBolt, but still remained 100% for both ClBolt and PCBolt. Although the detector in some cases was confused in classifying FCBolt and PCBolt, the overall accuracy of the detector was quite high at 96.3%.

**Figure 13:**
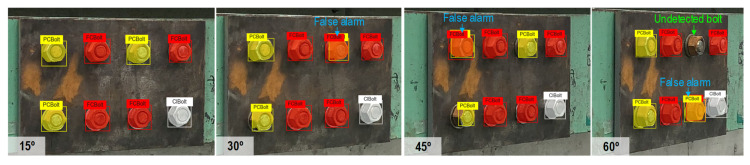
Visualization of corroded bolt detection results at different capturing angles.

As shown in Figure 12c,d, when the capturing angle increased to 45° and 60°, the overall accuracy of the detector was reduced to 94.05% and 87.64%, respectively. At the same time, the prediction accuracy for FCBolt dropped slightly from 93.02% to roughly 90.91%, respectively, and the accuracy for PCBolt fell from 96.67% to 82.35%, respectively. Figure 13 shows the representative corroded bolt identification results for different perspective angles. It is obvious that the detector was accurate in classifying FCBolt, with a desired accuracy of 96.67% for a perspective angle of up to 45°. 

**Figure 14:**
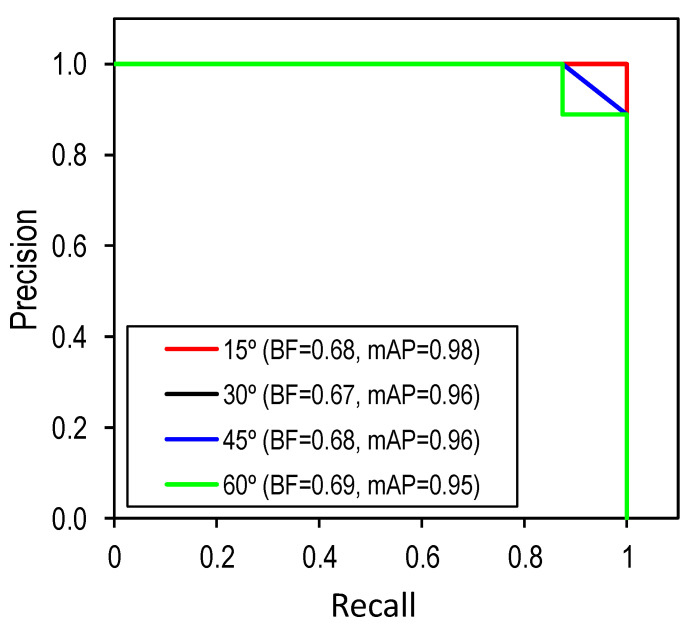
P-R curve, BF and mAP for different capturing angles.

Figure 14 shows the P-R curves, the BF and mAP values of the corroded bolt detector for different perspective distortions. The BF scores at four perspective distortions range between 0.67 and 0.69. It is found that the obtained BF scores are quite low because the detector identified most of the bolt washers, which were not pre-defined. Further, the mAP scores lie in the range of 0.95 to 0.98. The mAP was ignorably lower when the perspective angle was increased.

## 6. Concluding Remarks

In this study, the Mask-RCNN model was developed for the early identification of partially corroded bolts in steel structures. In the model, the Resnet50 integrated with the feature pyramid network was used as the backbone for feature extraction. To overcome the issue of limited training data, a four-step data synthesis method was newly proposed for the Mask-RCNN corroded bolt detector. To evaluate the accuracy of the proposed method, the experiment was conducted on a lab-scale steel structure for which corroded bolts were detected under varying capturing distances and perspectives. 

From the experimental evaluation, the following concluding remarks are drawn:

(1) Clean bolt, partially and fully corroded bolts along with their corresponding masks were autonomously created by the proposed data synthesis method. The Mask-RCNN-based detector was successfully trained using the generated datasets.
(2) The trained detector was accurate for corroded bolts in the tested structure. The corroded bolts and their corrosion levels were detected with the most desired accuracy of 96.3% for the 1.0-m capturing distance and 97.5% for the 15° perspective angle.
(3) The number of pixels for the test image of the bolt connection should not be less than 263K to ensure the accuracy of the bolt identification results.

In comparison to a previous study [24], it was found that the autonomous image generation and label annotation method significantly reduced labor cost and working time. Moreover, the Mask-RCNN-based detector could identify partially corroded bolts by distinguishing them from fully corroded bolts. Regarding the effect of capturing angles, the proposed detector could achieve an accuracy up to 97.5% at a 15° perspective angle. For the varying capturing distances test, the accuracy of both models shows a tendency to decline along with the capturing distance.

The proposed data synthesis method is autonomous and fast. Thus, it is promising for computer-vision-based damage detection in practice using standard and high-quality datasets at a low computational cost. In particular, the method could be used to simulate damage (i.e., cracks, spalling, defects, corrosion, etc.) on the structure surfaces that have rarely been caught during their operation period. Furthermore, the generated synthetic data could be used as training data sets for multi-targets, such as semantic segmentation, object detection, etc., with only minor changes in the use of programing language.

In some cases, the detector could not distinguish the partially and fully corroded bolts. This might be due to the presence of bolt washers, as those were missed from the generated training datasets. Therefore, research needs remain (1) to improve the accuracy of the developed corroded bolt detector, (2) to compare the performance of the detector on different data types with and without using the pre-trained model, (3) to investigate the accuracy of the model by changing parameters such as the sizes of the training images, sizes of the corroded bolt foregrounds, the flashlight of cameras, and the lights of the environment, and (4) to estimate the corrosion areas of detected bolts.

## Figures and Tables

**Table 1:** Detailed layers and operators of modified Resnet50-FPN.

No	Type	Depth	Filter Size	Stride	Padding	Output Image Size
1	Input	3	-	-	-	[w × h] *
2	Conv	64	7 × 7	2	3	[w/2 × h/2]
3	Batch norm	-	-	-	-	[w/2 × h/2]
4	ReLu	-	-	-	-	[w/2 × h/2]
5	Max pool	64	3 × 3	2	2	[w/4 × h/4]
6/8/10/12. Conv block	Conv-1	64/128/256/512	1 × 1	1/2/2/1	0/0/2/0	[w/4 × h/4] for block #6 [w/8 × h/8] for block #8 [w/16 × h/16] for block #10 [w/32 × h/32] for block #12
	BN-1	-	-	-	-	
	ReLu-1	-	-	-	-	
	Conv-2	64/128/256/512	3 × 3	1	1	
	BN-2	-	-	-	-	
	ReLu-2	-	-	-	-	
	Conv-3	256/512/1024/2048	1 × 1	1	0	
	BN-3	-	-	-	-	
	ReLu-4	-	-	-	-	
	Conv-4	256/512/1024/2048	1 × 1	1/2/2/1	0/0/2/0	
	BN-4	-	-	-	-	
7/9/11/13. ID Block	Conv-5	64/128/256/512	1 × 1	1	0	[w/4 × h/4] for block #7 [w/8 × h/8] for block #9 [w/16 × h/16] for block #11 [w/32 × h/32] for block #13
	BN-5	-	-	-	-	
	ReLu-5	-	-	-	-	
	Conv-6	64/128/256/512	3 × 3	1	1	
	BN-6	-	-	-	-	
	ReLu-6	-	-	-	-	
	Conv-7	256/512/1024/2048	1 × 1	1	0	
	BN-7	-	-	-	-	
	ReLu-7	-	-	-	-	

* w and h are the width and height of an image.

**Table 2:** Details of image properties in the dataset.

	Raw Images		Foreground (FG)			Sub-Background (SBG)	Dataset		
	Image	BG	FG-1	FG-2	FG-3		D1	D2	D3
Number of images	568	9	566	332	865	414 *	1875	1875	1875
Size	3024 × 4032 × 3		34 × 42 × 3–173 × 187 × 3			512 × 512 × 3	512 × 512 × 3		
Color	RGB **		RGBA **			RGB	RGB		
Format	.jpeg		.png			.jpeg	.jpeg		

* 414 images of SBG per 9 background, ** RGB(A): red, green, blue, (alpha) color.

## Data Availability

Data available on reasonable request from the corresponding author.
